# Clinical analysis of 312 patients with stage IB1-IIA2 cervical squamous cell carcinoma and research on the influencing factors of postoperative recurrence

**DOI:** 10.1186/s12905-023-02159-9

**Published:** 2023-02-23

**Authors:** Jia Zeng, Jing Zuo, Ning Li, HongWen Yao, YuanYuan Zhang, YuXi Zhao, TianTian Wang, Lin Xiu, Jian Li, Jing Yu, LeiLei Liang, LingYing Wu

**Affiliations:** Department of Gynecologic Oncology, National Cancer Center/National Clinical Research Center for Cancer/Cancer Hospital, Chinese Academy of Medical Sciences and Peking Union Medical College, No. 17 of Panjiayuan Nanli, Chaoyang District, Beijing, 100021 China

**Keywords:** Cervical cancer, Radical hysterectomy, Human papillomavirus, Viral load, Recurrence

## Abstract

**Objective:**

To investigate the influencing factors of the recurrence of IB1-IIA2 cervical squamous cell carcinoma after surgical treatment, and to explore the relationship between high-risk human papillomavirus (HR-HPV) infection and postoperative cervical squamous cell carcinoma recurrence.

**Methods:**

Patients (n = 312) diagnosed with stage IB1-IIA2 cervical cancer and treated by radical hysterectomy and lymphadenectomy at this hospital were accrued between January 2014 and December 2016. The clinical data of these patients were analysed, and the association among clinicopathological factors, the association among clinicopathological factors, HPV infection and recurrences was investigated through Cox regression.

**Results:**

The median follow-up time was 59.2 months (with a range of 14–77.9 months). The pre-operative HPV infection rate was 85.3% (266/312), and 74 patients had a high level of HPV-DNA (> 5 × 10^6^ copy number / 10^4^ cells). Twenty-nine patients had a postoperative persistent high level of HPV-DNA (9.3%). On multivariate analysis, deep 1/3 stromal invasion (hazard ratio [HR] 114.79, 95% confidence interval [CI] 2.821–4670.111, *p* = 0.012*) and postoperative persistence of high HPV-DNA levels within 12 months (HR 269.044, 95% CI 14.437–5013.754, *p* < 0.001*) and 24 months (HR 31.299, 95% CI 1.191–822.215, *p* = 0.039*) were associated with a higher local recurrence rate.

**Conclusion:**

Continuous high HPV-DNA levels within 24 months of an operation and deep 1/3 interstitial infiltration were independent risk factors for local recurrences of cervical cancer.

## Highlights

1. The relationship between postoperative HPV infection and cervical cancer recurrences was explored for the first time.
2. The genotypes and viral loads of 15 types of HR-HPV were determined.
3. Continuous HR-HPV positivity after surgery and deep 1/3 interstitial infiltration were risk factors for the recurrence of cervical cancer.

## Introduction

Cervical cancer is the fourth most common cancer among women worldwide. In 2018, there were an estimated 570,000 new cases and 311,000 deaths worldwide, including 106,000 cases and 48,000 deaths in China alone [1]. China’s contribution to the global total amounted to 18.6% of new cases and 15.4% of deaths [2]. The cervical cancer screening strategy is based on the human papillomavirus (HPV) test and the Papanicolaou test [3]. Postoperative recurrence is the main cause of death in patients with cervical cancer. Studies show that the recurrence rate of cervical cancer five years after surgery can be as high as 13.40% [4]. Despite a decrease in cervical cancer occurrences in developed countries due to screening programs, the frequency of this disease in developing nations continues to increase at an alarming rate, at least partly due to insufficient HPV screening and follow-up [5]. Strict postoperative follow-up and early warnings are important to prevent the recurrence of cervical cancer after surgery.

A high-risk HPV (HR-HPV) infection is closely related to cervical cancer and its precancerous lesions, namely cervical intraepithelial neoplasia (CIN). Its sensitivity and negative predictive value in diagnosing cervical lesions can even be as high as 100%. Therefore, HR-HPV detection is currently attracting much attention in cervical lesion screenings [6]. There are significant differences in clinical treatment strategies and prognoses among patients with different degrees of CIN. Finding sensitive indicators that can reflect the degree of CIN and prognosis is the focus of clinical attention [7]. Tumour size, stromal invasion, lymphovascular space involvement (LVSI), pathologically confirmed lymph node metastases, extensions into parametrial tissue and positive surgical margins are predictors of recurrence after primary surgery [8]. Some studies suggest that the HPV viral load is closely related to the degree of CIN [9]. Moreover, research by Yu et al. [10] found that the HPV viral load can be at a low level in patients with cervical cancer but at a high level in chronic cervicitis and even among healthy women. In addition, there are few research reports on the relationship between HR-HPV viral loads and the clinical outcomes of patients with CIN. It is still controversial whether an HR-HPV viral load can be used as the evaluation standard of the pathological degree and prognosis of clinical CIN [11], particularly the postoperative HPV viral load.

In this study, the clinical data and outcomes of 312 patients with stage IB1-IIA2 cervical squamous cell carcinoma who underwent surgical treatment were analysed, and the potential influencing factors affecting postoperative recurrence were studied to provide a new perspective for evaluating the risk of recurrence.

## Materials and methods

### Patient characteristics

Data on patients with stage IB1-IIA2 [12] cervical cancer treated in this hospital from January 2014 to December 2016 were analysed retrospectively. The inclusion criteria included the following: (1) patients were treated by radical hysterectomy (RH) with pelvic ± para-aortic lymphadenectomy; (2) they accepted pre- and postoperative HPV tests by an HPV real-time polymerase chain reaction (qRT-PCR) kit (Z-ME-0100-50, Liferiver, Shanghai, China); (3) patients had squamous cell carcinoma; (4) they had normal mental cognition; (5) they provided complete follow-up data for at least 14 months. The exclusion criteria were as follows: (1) patients had comorbidity with malignant tumours in other parts of the body, severe organ dysfunction or immune system diseases; (2) they had received drug treatment or chemoradiotherapy before enrolment; (3) they had a previous history of cervical or uterine surgery or HPV vaccination; (4) pregnant or lactating women; (5) the follow-up was not completed as required, or the follow-up data were incomplete. This study was conducted following the Declaration of Helsinki (2013) and approved by the Ethics Committee of the National Cancer Center/Cancer Hospital, Chinese Academy of Medical Sciences and Peking Union Medical College.

### Treatment and follow-up

Radical hysterectomy with pelvic node dissection are the primary methods of treatment for patients with early-stage cervical cancer. The present investigation highlighted that in referral centres, the shift from minimally invasive to open RH did not influence 90-day surgery-related morbidity [13, 14]. All patients were treated by RH and pelvic ± para-aortic lymphadenectomy, including laparotomy and laparoscopy. The ipsilateral or bilateral ovaries were preserved for patients younger than 45 years old. Patients with LVSI, a positive surgical margin, stromal invasion or lymph node metastasis received either adjuvant radio/chemotherapy or concurrent radio/chemotherapy (CCRT). The total dosage of radiotherapy was 45–50 Gy of cisplatin (40 mg/m^2^), which was administered during CCRT on a weekly basis. The volume of external beam radiotherapy covered the region of the previous gross disease, parametrial space, uterosacral ligaments, three centimeters of tissue at the proximal end of the vagina and all pelvic nodal volumes at risk. The follow-up period was the duration from the date of the surgical operation to either the end of 30 December 2020 or the date of recurrence.

### Data collection

The data of all patients were retrospectively collected, including pre-diagnosis, the degree of the disease, the treatment methods adopted and postoperative follow-up.

### Human papillomavirus-deoxyribonucleic acid testing

All patients consented to receiving the HPV-deoxyribonucleic acid (DNA) test within one month pre-operatively and at multiple time points during the postoperative follow‐up period. The patients were instructed to avoid vaginal douching three days before the examination, abstain from sex the day before and avoid having the examination during their menstrual period. Patients were placed into the lithotomy position during the examination, and after the cervix was exposed, it was beneficial for the special collector to enter the cervix for sampling. The brush was placed in the cervix, rotated clockwise for three to five times, placed in a special storage bottle, sealed and sent for inspection. ThinPrep cytology specimens were tested by the HPV qRT-PCR kit (Z-ME-0100-50, Liferiver, Shanghai, China), which detects viral DNA by nucleic acid hybridisation with a pooled probe set for 15 HR-HPV genotypes (HPV16, 18, 31, 33, 35, 39, 45, 51, 52, 56, 58, 59, 66, 68 and 82). The nucleic acid was extracted by an automatic nucleic acid extracting machine (Autrax Bio-system®, Liferiver, Shanghai, China). All PCR were performed by a qRT-PCR thermocycler (SLAN-96P real-time PCR system, Shanghai Hongshi Corp, China) with a detection range from 5 × 10^2^ to 5 × 10^7^ copies/104 cells. All data collected were based on the official report by the laboratory test centre of this hospital.

### Human papillomavirus infection status

Patients infected by two or more HPV genotypes were defined as having coinfection. An HPV-DNA level > 5 × 10^6^ copies/10^4^ cells was determined to be high-level. Pre-operatively, all patients were classified into negative, low-level or high-level groups. According to the postoperative clearance time, patients were classified into five groups: pre- and postoperative negative, cleared within 12 months, cleared within 12–24 months, cleared after 24 months and uncleared. Based on the time of high HPV-DNA persistence, patients were classified into the groups of postoperative persistence within 12 months, 12–24 months and longer than 24 months.

### Statistics

Using SPSS 26.0 software, the measurement data are described by mean ± standard deviation. The comparison between groups was described with a one-way analysis of variance. The counting data were expressed in the number of cases (percentage) [n (%)]. Either the chi-square or Fisher's exact test was used for comparison between groups. Spearman rank correlation was used in the correlation analysis. The factors affecting postoperative recurrence were explored by univariate analysis, and the meaningful factors were then included in multivariate Cox regression analysis to further explore the independent risk factors affecting postoperative recurrence. The inspection level α = 0.05, *p* < 0.05 was statistically significant.

## Results

### Characteristics of patients

The data of 1,030 patients from January 2014 to December 2016 were reviewed, of which 312 met the inclusion criteria. All 312 patients were treated by RH with lymphadenectomy, while 123 patients (39.4%) with pathological risk factors also received postoperative adjuvant radio/chemotherapy. The average age of the 312 patients was 47 (with a range of 25–73) years old. Pathologists confirmed no patients had parametrial invasions or positive resection margins. The average follow-up time was 59.2 months (with a range of 14–77.9 months). The characteristics of the patients are shown in Table 1.

**Table 1:** Characteristics of patients (n = 312)

Characteristic	No. (%)
*Age (yr)*	
Median (range)/year-old	47 (25–73)
< 50	198 (63.5)
≥ 50	114 (36.5)
*FIGO stage(2014)*	
IB1	226 (72.4)
IB2	45 (14.4)
IIA1	27 (8.7)
IIA2	14 (4.5)
*Histological Grade*	
Low	32 (10.3)
Moderate	142 (45.5)
High	138 (44.2)
*Tumor size*	
< 4 cm	254 (81.4)
≥ 4 cm	58 (18.6)
*Stromal invasion*	
Superficial 1/3 and middle 1/3	284 (91)
Deep 1/3	28 (9)
*LVSI*	
No	190 (60.9)
Yes	122 (39.1)
*Lymph node metastasis*	
No	279 (89.4)
Yes	33 (10.6)
Parametrial invasion	0
Positive resection margin	0
*Treatment*	
RH	189 (60.6)
RH + Adjuvant radio therapy	123 (39.4)
RH + concurrent	33 (26.8)
radio(chemo)therapy(CCRT)	90 (73.2)
*Surgery type*	
LRH	197 (63.1)
ARH	115 (36.9)
Local recurrence	7 (2.2)
Distant metastasis	5 (1.6)
Death	4 (1.3)
Median Follow-up (range)/months	59.2 (14–77.9)

*FIGO* International Federation of Gynecology and Obstetrics, *LVSI* lymphovascular space invasion, *RH* radical hysterectomy, *LRH* laparoscopic radical hysterectomy, *ARH* abdominal radical hysterectomy

### Clinical analysis of patients

Among the 312 patients, 266 patients (85.3%) were HPV-positive before the operation, including 227 patients (72.8%) infected by HPV-16/18 and 39 (12.5%) patients infected by 13 other genotypes of HR-HPV. There were 192 patients in the low HPV-DNA level group and 74 patients in the high HPV-DNA level group (Table 2).

**Table 2:** Clinical data of 312 patients before and after operation

Characteristic	No. (%)
*Pre-operative HPV infection status*	
Negative	46 (14.7)
Single genotype	214 (68.6)
Multiple genotypes	52 (16.7)
HPV-16/18 positive	227 (72.8)
Other hr-HPV	39 (12.5)
*Pre-operative HPV-DNA level*	
Negative	46 (14.7)
Low level	192 (61.5)
High level	74 (23.7)
*Post-operative HPV infection status*	
Negative	224 (73.1)
Single genotype	61 (19.6)
Multiple genotypes	27 (8.7)
HPV-16/18 positive	16 (6)
Other hr-HPV	72 (27.1)
*Post-operative HPV-DNA level*	
Persistent negative	224 (71.8)
Low level	59 (18.9)
Persistent High level (< 12 m)	11 (3.5)
Persistent High level (12-24 m)	8 (2.6)
Persistent High level (> 24 m)	10 (3.2)

All patients underwent a postoperative HPV test every 3–12 months. Among the 266 pre-operative HPV-positive patients, 209 were HPV-negative within 12 months, during 12–24 months and 4 after 24 months; 32 patients had persistent HPV infection at the end of the 24 months. Additionally, 270 (86.5%) patients were cleared of HPV 24 months post-operation. Regarding the postoperative level of HPV-DNA, 59 patients had a low level of HPV-DNA, and 29 patients had a persistently high level, including 11 patients within 12 months, 8 patients within 12–24 months and 10 patients longer than 24 months (Table 2).

### Clinical information and HPV infection in seven recurrent patients

The median follow-up time was 59.2 months (with a range of 14–77.9 months). Seven patients had a local recurrence and five patients had distant metastasis. The average local recurrence time was 12 months (with a range of 6–18 months). Three patients died of an uncontrolled local recurrence, while one died of liver metastasis. The five-year local recurrence-free survival rate was 97.8% (305/312 patients), and the five-year overall survival rate was 98.7% (308/312). The recurrence status of seven patients is demonstrated in Table 3.

**Table 3:** Clinical information and HPV infection status of 7 recurrent patients

Patient No	Surgery date	Date of recurrence confirmed	Recurrence location	Postoperative HPV infection genotype(s)	Postoperative HPV viral load(HPV-DNA level)
1	1/28/2015	10/4/2015	Pelvic	HPV-16	5 × 10^6^ copies/10^4^ cells
2	5/4/2015	5/19/2016	Pelvic	HPV-56	5 × 10^6^ copies/10^4^ cells
3	11/19/2014	3/22/2015	Pelvic	HPV-16	5 × 10^6^ copies/10^4^ cells
4	6/27/2016	6/30/2017	Pelvic	Negative	0
5	5/16/2014	11/17/2014	Pelvic	HPV-16	5 × 10^7^ copies/10^4^ cells
6	7/6/2016	1/13/2018	Pelvic	HPV-16	5 × 10^7^ copies/10^4^ cells
7	7/1/2015	11/6/2016	Pelvic	Negative	0

### Analysis of influencing factors of recurrence

**Fig. 1:**
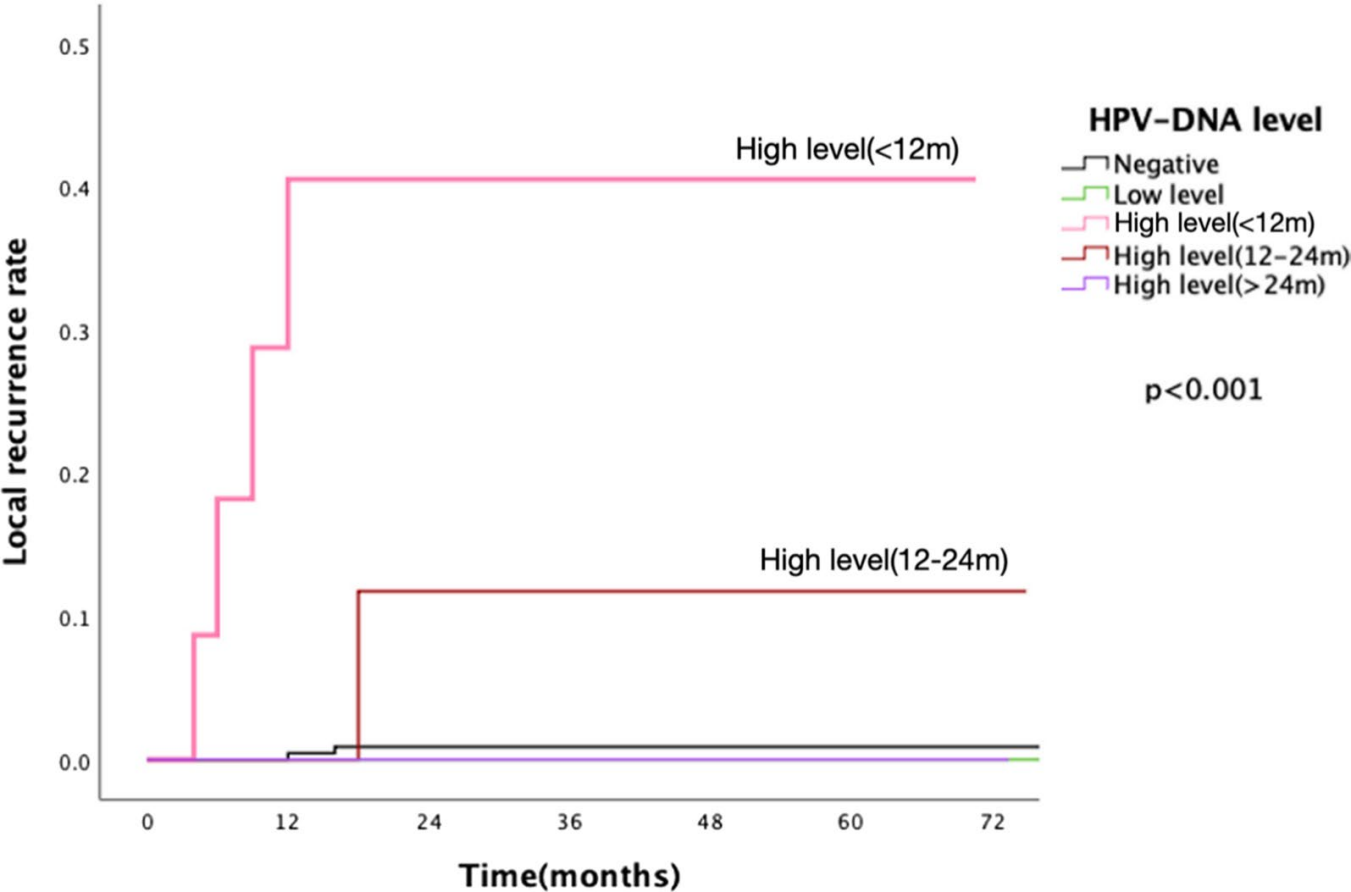
Risk of Local recurrence according to the postoperative HPV-DNA level

On univariate analysis, lymph nodes metastasis (hazard ratio [HR] 6.459, 95% confidence interval [CI] 1.445–28.859, *p* = 0.014*) and postoperative persistent high levels of HPV-DNA within 12 months ((HR 55.5, 95% CI 8.831–348.792, *p* < 0.01) and 12–24 months (HR 13.875, 95% CI 1.137–169.359, *p* = 0.039*) were associated with a higher local recurrence. Pre-operative HPV-DNA levels and HPV-16/18 infections had no impact on the local recurrence rate. Multivariate Cox regression analysis showed that the deep 1/3 stromal invasion (HR 114.79, 95% CI 2.821–4670.111, *p* = 0.012*), postoperative persistent high levels of HPV-DNA within 12 months (HR 269.044, 95% CI 14.437–5013.754, *p* < 0.001*) and 12–24 months (HR 31.299, 95% CI 1.191–822.215, *p* = 0.039*) were the independent risk factors for local recurrence (Table 4, Fig. 1).

**Table 4:** Univariate analysis and multivariate analysis to evaluate the influencing factors of local recurrence

	N	Univariate analysis (p-value)			Multivariate analysis(p-value)		
		HR	95%CI	p	HR	95%CI	p
*Tumor size*							
≥ 4 cm vs < 4 cm	254 vs. 58	0.036	0–128.503	0.426	0.227	0–4,490,352.617	0.863
*LVSI*							
Yes vs. No	122 vs. 190	2.082	0.466–9.304	0.333	0.655	0.053–8.046	0.741
*Stromal invasion*							
Deep 1/3 vs. superficial 1/3 and middle 1/3	28 vs. 284	4.106	0.796–21.164	0.091	114.79	2.821–4670.111	0.012*
*Lymph nodes metastasis*							
Yes vs. No	33 vs. 279	6.459	1.445–28.859	0.014*	22.706	0.863–597.154	0.061
*FIGO stage(2009)*							
IB2-IIA2 vs. IB1	86 vs. 226	0.383	0.046–3.178	0.373	0.006	0–3.457	0.116
*Surgery type*							
LRH vs. ARH	197 vs. 115	2.33	0.512–10.602	0.274			
*Pre-operative infection status*							
HPV-16/18 vs. Negative	227 vs. 46	0.844	0.051–13.96	0.906			
Other 13 Types vs. Negative	39 vs.. 46	0.856	0.097–7.529	0.888			
*Pre-operative HPV-DNA level*							
Low viral load vs. Negative	192 vs. 46	0.474	0.042–5.34	0.545			
High viral load vs. Negative	74 vs. 46	2.571	0.278–23.749	0.405			
*Post-operative clearance time (Initial HPV positive)*	N = 266						
Cleared (within 12 m) vs. Pre- and Post-operative Negative	209 vs. 38	0.177	0.011–2.893	0.224			
Cleared (12–24 m) vs. Pre- and Post-operative Negative	21 vs. 38	0	0–	0.998			
Cleared (after 24 m) vs. Pre- and Post-operative Negative	4 vs. 38	0	0–	0.999			
Uncleared vs. Pre- and Post-operative Negative	32 vs. 38	6.852	0.756–62.061	0.087			
*Post-operative HPV-DNA level*							
Low level vs. Persistent negative	59 vs. 224	0	0–	0.997	0	0–	0.997
High level (< 12 m) vs. Persistent negative	11 vs. 224	55.5	8.831–348.792	< 0.001*	269.044	14.437–5013.754	< 0.001*
High level (12-24 m) vs. Persistent negative	8 vs. 224	13.875	1.137–169.359	0.039*	31.299	1.191–822.215	0.039*
High level (> 24 m) vs. Persistent negative	10 vs. 224	0	0–	0.999	0	0–	0.999

*LVSI* lymphovascular space invasion, *FIGO* International Federation of Gynecology and Obstetrics, *LRH* laparoscopic radical hysterectomy, *ARH* abdominal radical hysterectomy

*p < 0.05 was statistically significant

## Discussion

According to the 2020 comprehensive global cancer statistics published by the International Agency for Research on Cancer, gynaecological malignancies accounted for 16.5% of an estimated 8.2 million overall new cancer cases in women. Gynaecological cancers represent an ongoing source of concern due to their high incidence and cancer-related mortality [15].

Early-stage patients had a better prognosis than advanced-stage patients (IIB-IV) [16–18], but they could have a recurrent disease occasionally. The prognosis of early-stage cervical cancer was related to pathological risk factors, including lymph node metastasis, positive resection margin, tumour size, parametrial invasion, deep stromal invasion and LVSI [19]. The present study showed that deep 1/3 stromal invasion was an independent risk factor of local recurrence (HR 114.79, 95% CI 2.821–4670.111, *p* = 0.012), which was in accordance with the former study. In addition to pathological risk factors, some studies focused on other factors, such as HPV infection status, including genotypes and viral load.

At present, more than 200 genotypes of HPV have been isolated and the carcinogenicity of different HPV genotypes varies widely [20, 21]. In the present study, the HPV test was based on qRT-PCR, which confirmed that 85.3% (266/312) of patients were HPV-positive. Among the pre-operative HPV-positive patients, 85.3% of them were HPV-16/18 positive, concordant with other studies (70–90%) [22–26]. And among the seven patients with a postoperative recurrence, the genotype of HPV infection in four was HPV-16.

Chen et al. reported that women with viral loads ≥ 5.22 copies/10^4^ cells might have a higher risk for residual lesions (*p* = 0.007). Furthermore, except for HPV-31/33, the viral loads of HP-16/52/58 showed significant differences in the number of residual lesions (*p* = 0.016, *p* < 0.001, *p* < 0.001). Thus, they concluded that HR-HPV viral loads could be a reliable predictor of residual lesions [27]. The present study showed that the pre-operative HPV viral load was not associated with the local recurrence rate (*p* > 0.05). Similar findings have been reported by previous studies [28, 29]. However, the present study showed that the postoperative persistence of high HPV-DNA levels within 12 months (HR 269.044, 95% CI 14.437–5013.754, *p* < 0.001*) and 12–24 months (HR 31.299, 95% CI 1.191–822.215, *p* = 0.039*) were independent risk factors for a higher local recurrence. Although this conclusion is inconsistent with Mahantshetty et al. [30], both studies used PCR-based HPV tests. Research reports show that using the Digene Hybrid Capture 2 (HC2) HPV DNA test and fluorescence in situ hybridisation (FISH) with a chromosome probe to TERC (3q26) to detect and evaluate HPV also have high sensitivity and specificity [31]. The HPV qRT-PCR kit (Z-ME-0100-50, Liferiver, Shanghai, China) was approved for clinical use by the China Food and Drug Administration. Therefore, more data on postoperative HPV infection statuses, including genotypes and viral loads, through this test method will be available in the future for analysis.

Cervical intraepithelial neoplasia is the precursor of cervical cancer [32]. Adcock et al. [33] reported that the risk of developing high-grade CIN depended on both genotypes and viral loads of HPV. There is an association between diagnosis of CIN3, presence of HR-HPV types, positive endocervical margins, HPV persistence, and the omission of HPV vaccination after conisation and the risk of developing cervical dysplasia persistence/recurrence [34]. Therefore, accurate measurement of the HPV genotype and viral load is essential for the effective diagnosis of cervical cancer and postoperative recurrences of cervical cancer.

This study has some highlights. On the one hand, the genotypes and viral loads of 15 HR-HPV types were evaluated using a highly sensitive and specific HPVqRT-PCR kit. However, this study is the first to examine the relationship between postoperative HPV infection and recurrence. In addition, this study is a statistical analysis based on clinical data, which has high clinical significance. Nevertheless, there are also some limitations to this study. First, it was retrospective and lacked prospective interventional studies to verify. Second, statistics on postoperative complications were lacking, which is also a potential factor that has a great impact on the outcome. Thus, future prospective studies will be conducted to evaluate the prognostic value of postoperative HPV viral loads in IB1-IIA2 cervical cancer patients.

## Conclusion

This study shows that a continuously high HPV-DNA level within 24 months after the operation and deep 1/3 interstitial infiltration are independent risk factors for a local recurrence of cervical cancer. Therefore, early and accurate postoperative HPV-DNA detection is useful in evaluating the risk of postoperative recurrence for patients.

## Data Availability

All data generated or analyzed during this study are included in this article.
